# Testing the performance of a fragment of the COI gene to identify western Palaearctic stag beetle species (Coleoptera, Lucanidae)

**DOI:** 10.3897/zookeys.365.5526

**Published:** 2013-12-30

**Authors:** Karen Cox, Arno Thomaes, Gloria Antonini, Michele Zilioli, Koen De Gelas, Deborah Harvey, Emanuela Solano, Paolo Audisio, Niall McKeown, Paul Shaw, Robert Minetti, Luca Bartolozzi, Joachim Mergeay

**Affiliations:** 1 Research Institute for Nature and Forest, Gaverstraat 4, B-9500 Geraardsbergen, Belgium; 2 Department of Biology and Biotechnology “Charles Darwin”, Sapienza - University of Rome, via A. Borelli 50, I-00161 Rome, Italy; 3 Natural History Museum, Entomological section, Corso Venezia 55, I-20121 Milano, Italy; 4 Royal Belgian Institute of Natural Sciences, Vautierstraat 29, B-1000 Brussels, Belgium; 5 School of Biological Sciences, Royal Holloway, University of London, Egham, Surrey, UK; 6 Institute of Biological, Environmental and Rural Sciences (IBERS), Aberystwyth University, Penglais, Aberystwyth, UK; 7 7 Avenue Marc Sangnier, 13600 La Ciotat, France; 8 Natural History Museum, Zoological Section “La Specola”, via Romana 17, 50125 Firenze, Italy

**Keywords:** *Lucanus* spp., Stag beetle, Western Palaearctic, DNA barcoding, COI

## Abstract

The taxonomy of stag beetles (Coleoptera: Lucanidae) remains challenging, mainly due to the sexual dimorphism and the strong allometry in males. Such conjecture confounds taxonomic based conservation efforts that are urgently needed due to numerous threats to stag beetle biodiversity. Molecular tools could help solve the problem of identification of the different recognized taxa in the “*Lucanus cervus* complex” and in some related Palaearctic species. We investigated the potential use of a 670 bp region at the 3’ end of the mitochondrial cytochrome *c* oxidase subunit I gene (COI) for barcoding purposes (different from the standard COI barcoding region). Well resolved species and subspecies were *L. tetraodon*, *L. cervusakbesianus*, *L. c. laticornis*, as well as the two eastern Asian outgroup taxa *L. formosanus* and *L. hermani*. Conversely, certain taxa could not be distinguished from each other based on K2P-distances and tree topologies: *L. c. fabiani* / *L. (P.) barbarossa*, *L. c. judaicus* / an unknown *Lucanus* species, *L. c. cervus* / *L. c. turcicus* / *L. c. pentaphyllus* / *L. (P.) macrophyllus* / *L. ibericus*. The relative roles of phenotypic plasticity, recurrent hybridisation and incomplete lineage sorting underlying taxonomic and phylogenetic discordances are discussed.

## Introduction

Lucanidae Latreille, 1804 is a family of Coleoptera showing in most species pronounced sexual dimorphism and strong external morphological allometry in males. The species of the Holarctic and Oriental distributed genus *Lucanus* Scopoli, 1763 are renowned for the striking appearance of the males. With their large body size and prominent mandibles, the male stag beetles are very popular among amateur entomologists and as terrarium pets, mainly in Japan. Currently, there are more than 90 *Lucanus* species described, however, validity of these designations is considered questionable in many cases. Sexual dimorphism and size variation complicate the taxonomy (Didier and Séguy 1953, Clark 1977, Harvey and Gange 2006), as does the lack of informative phenotypic characters among larvae. Consequently, their classification has changed over time and is still under discussion. In this study we focus on taxa of the *Lucanus* species in the western Palaearctic.

The genus *Lucanus* is subdivided into the subgenera *Lucanus* sensu stricto and *Pseudolucanus* Hope & Westwood, 1845. Members of the latter have a peculiar stout body and substantial analogy of morphology that makes it quite easy to distinguish them from members of the subgenus *Lucanus* (Planet 1899). The male mandibles of *Pseudolucanus* are sickle shaped, their internal edge has a single denticle in most species (*Lucanus* has small denticles and one large denticle) and the apex is usually simple (*Lucanus* is mostly bifid) (Planet 1899, Baraud 1993). Furthermore, the integument of *Pseudolucanus* is relatively smooth with scattered and superficial punctuation whereas it is more stippled in *Lucanus*. Also, the sides of the pronotum of *Pseudolucanus* are strongly sinuate before the posterior angles (Baraud 1993). Previous studies (Didier and Séguy 1953, Benesh 1960, Krajcik 2001, Bartolozzi and Sprecher-Uebersax 2006, Hallan 2008, Fujita 2010) describe between four and seven species of *Lucanus* in western Palaearctic: i.e. *Lucanus (Lucanus) cervus* (Linnaeus, 1758), *Lucanus (Lucanus) ibericus* Motschulsky, 1845, *Lucanus (Lucanus) orientalis* Kraatz, 1860, *Lucanus (Lucanus) tetraodon* Thunberg, 1806, *Lucanus (Pseudolucanus) barbarossa* Fabricius, 1801, *Lucanus (Pseudolucanus) busignyi* Planet, 1909 and *Lucanus (Pseudolucanus) macrophyllus* Kraatz, 1860.

The distribution of many of these taxa remains poorly resolved, however, we can consider some of them as endangered. The practice of removing old trees and dead wood in past and current forest management, has had detrimental effects on this group of saproxylic beetles (Jansson and Coskun 2008, Nieto and Alexander 2010). Consequently, the loss of habitat might have reduced the range of some taxa, especially the Mediterranean taxa where deforestation started a few millennia ago (Jansson and Coskun 2008, Buse et al. 2010). At least *Lucanus cervus cervus* seems to be able to cope with urbanisation (Thomaes et al. 2008) as long as the habitat turnover allows recolonisation (Thomaes 2009). In addition, beetle collecting can be considered as a threat when it goes hand in hand with large scale habitat destruction or when species rarity causes overexploitation (Holden 2007, Tournant et al. 2012). Another possible consequence of the international stag beetle trade is the introduction of non-native specimens which may cause genetic introgression (Goka et al. 2004) and transmission of parasites potentially pathogenic to native stag beetles (cf. Goka et al. 2004, Kanzaki et al. 2011). Unfortunately, legal protection is often missing or inadequate. The widely distributed *Lucanus cervus cervus* is protected by the Habitats Directive of the European Union from 1992 (Luce 1996) and is listed as “near threatened” in the Red Data list of Europe (Nieto and Alexander 2010). *Lucanus (Pseudolucanus) barbarossa* and *Lucanus tetraodon* are mentioned in the IUCN list, but are rated “of least concern” (IUCN 2012), while *Lucanus ibericus* is considered to be “vulnerable” within the EU 27 (Nieto and Alexander 2010).

More detailed information on the distribution and ecology of this species group is needed to get a clear view on their conservation status. But unless the problem of identification of European and West Asian *Lucanus* is solved, it becomes difficult to set specific conservation priorities, without which rare, neglected and endangered species or Evolutionarily Significant Units (ESUs) may be unrecognised and thus, not given adequate conservation prioritisation (Ryder 1986, Waples 1991, Moritz 1994a, Moritz 1994b, Fraser and Bernatchez 2001). Molecular tools could help identification of stag beetles. The mitochondrial cytochrome *c* oxidase subunit I (COI) is the most widely used gene in barcoding animals (Hebert et al. 2003). The barcoding practice entails the analysis of the DNA sequence of a part of this mitochondrial gene, typically between 600 and 900 bp. In this study, we investigated the use of the 3’ end of the COI gene, different from the standard barcoding region, for the identification of western Palaearctic *Lucanus* species and subspecies.

## Material and methods

### Taxonomy and morphology

*Lucanus cervus* has the widest geographical distribution in the genus and is very variable in form, size and colour (Harvey et al. 2011). Many subdivisions (i.e. subspecies or morphotypes) have been proposed and discussed. *Lucanus cervus cervus* (Linnaeus, 1758), the main subspecies found throughout Europe, has, in general, four lamellae on the antennal clubs and is typically bicoloured (black head and thorax, and reddish brown elytra and mandibles). *Lucanus cervus akbesianus* Planet, 1896 with generally six lamellae and large mandibles with a very open apical fork, inhabits southern Turkey and Syria. *Lucanus cervus turcicus* Sturm, 1843 also has a six lamellate club, but its mandibles are comparable to *Lucanus cervus cervus*. It is reported in Greece, Bulgaria and Trakya (European part of Turkey). Furthermore, *Lucanus cervus judaicus* Planet, 1902 with a four lamellate club and reddish brown colour, is found in the more eastern parts of Turkey and in northern Syria. *Lucanus cervus laticornis* Deyrolle, 1864, found in central and southern Turkey, has six long lamellae and the inner denticle of the mandibles is followed by two or three denticles. *Lucanus cervus fabiani* Mulsant & Godart, 1855 is an endemic taxon inhabiting southern France and shows a five lamellate club and slender, slightly curved mandibles with a simple apex and post-median denticle along with a few other denticles. The taxa *Lucanus fabiani* and *Lucanus pentaphyllus* Reiche, 1853 are listed as synonyms of *Lucanus cervus cervus* by Bartolozzi and Sprecher-Uebersax (2006), but *Lucanus fabiani* could well be considered as a valid species according to Boucher (unpublished data) while *Lucanus pentaphyllus* may represent a small form of *Lucanus cervus* with five lamellate clubs, a character that can also be found in *Lucanus cervus cervus*. Other taxa [*Lucanus tauricus* Motschulsky, 1845 (described from Crimea), *Lucanus poujadei* Planet, 1897 (Kurdistan), *Lucanus mediadonta* Lacroix, 1978 (Georgia) and *Lucanus pontbrianti* Mulsant, 1839 (France)], recognised by some authors as valid subspecies or simple synonyms, were not included in this study. Bartolozzi and Sprecher-Uebersax (2006) only list *Lucanus cervus* and *Lucanus judaicus* as separate subspecies. Hallan (2008) adds *akbesianus, fabiani, mediadonta, tauricus* and *Lucanus turcicus*, while Krajcik (2001) further includes *Lucanus pontbrianti* and *Lucanus laticornis*, although Schenk and Fiedler (2011) perceived *Lucanus laticornis* as a separate species. On the other hand, Didier and Séguy (1953) also list *Lucanus capreolus* Fuessly, 1775 (considered a small form of *Lucanus cervus*) and *Lucanus poujadei* while Fujita (2010) only recognises *Lucanus poujadei* but does not list *Lucanus tauricus* and *Lucanus mediadonta* or the [*pentaphyllus + fabiani + pontbrianti*] complex.

*Lucanus ibericus* can be found from Albania to Iran and is sometimes considered a synonym of *Lucanus orientalis*. Unlike *Lucanus cervus*, *Lucanus ibericus* is entirely reddish brown, has a pronotum without a smooth discal line, but with a sinuate posterior and distinct toothed posterior angles (non-sinuate pronotum and blunt angles in *Lucanus cervus*). The mandibles of the males, which are shorter than those of a typical male *Lucanus cervus* of equal size, can have an apex with two equal teeth or with the inner tooth fainted and a large internal denticle in the middle. In addition, *Lucanus ibericus* has six, rarely five, long lamellae on the antennal club.

*Lucanus tetraodon* described from France, Italy, North Africa, Albania and Greece, can be perceived as a central Mediterranean species. In contrast to *Lucanus cervus* and *Lucanus ibericus*, the basal denticle of the mandibles of *Lucanus tetraodon* is placed in the lower half. Like *Lucanus ibericus*, the pronotal sides have sharp posterior angles, but the pronotal disc misses the central smooth line. *Lucanus tetraodon* has six, occasionally five, lamellae on the antennal club. *Lucanus tetraodon* is by some authors subdivided in subspecies *Lucanus tetraodon argeliensis* Maes, 1995 in North Africa, *Lucanus tetraodon provincialis* Colas, 1949 in South France, *Lucanus tetraodon corsicus* Gautier des Cottes, 1860 in Corsica, *Lucanus tetraodon sicilianus* Planet, 1899 in Sicily and finally *Lucanus tetraodon tetraodon* Thunberg, 1806 elsewhere. In addition, specimens of problematic populations of *Lucanus cervus* from a series of localities in central Italy (northern Latium and Umbria), are known to exhibit apparently intermediate morphological characters between *Lucanus cervus* and *Lucanus tetraodon*, which are sympatric in these areas (Santoro et al. 2009).

The *Pseudolucanus* species all have six long lamellae forming the antennal club, their body is stout and entirely reddish or blackish brown. Included in this study are *Lucanus (Pseudolucanus) barbarossa* from the Iberian peninsula and the Maghreb, and *Lucanus (Pseudolucanus) macrophyllus* reported in south-west Turkey. Krajcik (2001) and Hallan (2008) list the latter as a subspecies of *Lucanus ibericus*. Schenk and Fiedler (2011) recently quoted populations of *Lucanus (Pseudolucanus) busignyi* in western Turkey, but this taxon is not included in this study.

### Taxon sampling and DNA extraction

A large number of entomologists was contacted to obtain material from the different taxa and from different regions. The samples included whole beetles, especially in regions where identification is problematic, as well as parts of a beetle, sometimes found as road kill or as prey leftovers from birds. Samples were dried and kept at room temperature or preserved in absolute ethanol. In total 76 samples were collected. The species identification was performed, using comparative material and available identification keys. Six samples from Israel and Lebanon could not be identified to species. These unidentified *Lucanus* specimens have a shape resembling in general the medium to small males of *Lucanus cervus akbesianus* but with a mandibular structure similar to that of *Lucanus cervus turcicus* (Zilioli et al. unpublished data). The tissue samples used for DNA extraction depended on what was available, but were mostly legs, which contain large muscles and are therefore rich in mitochondrial DNA (mtDNA). DNA was extracted from ground samples with the E.Z.N.A.® Forensic DNA Kit (Omega Bio-Tek), except for samples K1 and U6 (Table 1) from which DNA was extracted following the salting out procedure described by Aljanabi and Martinez (1997). The integrity of the extracted DNA was checked spectrophotometrically on a ND-1000 Nano-Drop (NanoDrop Technologies) and its quality on 1% agarose gels.

**Table 1.** List of samples included in the analysis. Primers used are denoted with ‘1’: C1-J-2183 and TL2-N-3014; ‘2’: LCint1F, LCint2F, LCint3F and LCint4F (for sample SB6 also the reverse primers were used); ‘3’: F - 5’ GGGGCATCAGTAGACCTAGC 3’ and R – 5’ TTCAGCAGGTGGTATTAGTTGG 3’.

Species / subspecies	Code	Primers	Haplotype	GenBank acc. no.	Country	Latitude	Longitude	Date of sampling	Type of conservation	Gender
*Lucanus cervus akbesianus*	UA1	1	UA1	KF737127	Turkey	37.721833, 30.828278		Jun 2010	ethanol	Female
	UA2	1	UA2	KF737128	Turkey	37.721833, 30.828278		Jun 2010	ethanol	Male
	UA3	1	UA3	KF737129	Turkey	37.721833, 30.828278		Jun 2010	ethanol	Male
	UA4	1	UA4	KF737130	Turkey	37.676200, 35.862100		2010	ethanol	Male
	UA5	1	UA5	KF737131	Turkey	37.676200, 35.862100		2010	ethanol	Male
	UX1	2	UX1	KF737132	Turkey	36.900000, 31.000000		Jun 2010	ethanol	Male
	U10	1	U10	KF737125	Turkey	37.721833, 30.828278		Jun 2010	ethanol	Male
	U11	1	U10	KF737126	Turkey	37.721833, 30.828278		Jun 2010	ethanol	Male
*Lucanus cervus cervus*	A1	1	A1	KF737071	Belgium	50.772652, 4.537656		Jul 2008	ethanol	Male
	A3	1	A3	KF737072	Belgium	50.736622, 4.331784		Jun 2009	ethanol	Female
	C1	2	C1	KF737093	Czech rep.	48.797935, 16.803576		May 2009	ethanol	Male
	D13	2	A3	KF737078	France	45.391800, 1.139310		Jul 2010	ethanol	Male
	D4	1	D4	KF737088	France	43.458090, 1.431787		Aug 2010	ethanol	Male
	D22	1	D22	KF737092	France	47.861145, 2.820327		2009	ethanol	Female
	F12	1	A3	KF737079	Greece	39.808333, 22.653889		Jun 2009	ethanol	Female
	F16	1	F16	KF737083	Greece	39.808333, 22.653889		Jun 2009	ethanol	Female
	F23	1	F23	KF737082	Greece	39.762333, 21.663281		Jun 2009	ethanol	Male
	G3	2	G3	KF737081	Hungary	47.701586, 18.834592		Jul 2009	ethanol	Female
	I2	1	I2	KF737084	Italy	45.779241, 8.732981		Jun 2009	ethanol	Male
	I3	1	A3	KF737080	Italy	45.779241, 8.732981		Jun 2009	ethanol	Male
	I4	1	I4	KF737085	Italy	45.779241, 8.732981		Jun 2009	ethanol	Male
	N3	1	N3	KF737086	Portugal	38.795900, -9.397390		Jul 2010	ethanol	Male
	O9	3	O9	KF737087	Romania	47.102400, 24.450700				
	S15	1	S15	KF737094	Spain	40.385100, -6.608460		Aug 2009	ethanol	Male
	S19	1	A3	KF737076	Spain	43.304009, -4.814970		Jul 2009	ethanol	Female
	V2	1	A3	KF737077	UK	52.028936, 1.067369		Aug 2009	dried	Female
	V26	3	V26	KF737091	UK	50.966300, -0.209294				
	V44	3	V44	KF737089	UK	51.260100, 0.844280				
	W9	2	W9	KF737090	Ukraine	49.826900, 36.325800		Jun 2007	dried	Male
	X1		X1	FJ606555	France	(Lin et al. 2011)				
*Lucanus cervus fabiani*	D11	1	D11	KF737121	France	43.195300, 5.753740		Jun 2010	ethanol	Male
*Lucanus cervus judaicus*	UJ1	1	UJ1	KF737112	Turkey	37.068100, 36.261600		Jul 2010	dried	Male
*Lucanus cervus laticornis*	UL2	1	UL2	KF737119	Turkey	36.875669, 30.457431		Jun 2007	ethanol	Male
	UL3	1	UL3	KF737120	Turkey	37.763600, 30.558900		1995	dried	Male
*Lucanus cervus pentaphyllus*	C2	1	A3	KF737075	Czech rep.	48.797935, 16.803576		May 2009	ethanol	Male
	F13	1	F13	KF737104	Greece	39.808333, 22.653889		Jun 2009	ethanol	Female
	I1	1	A3	KF737073	Italy	45.779241, 8.732981		Jun 2009	ethanol	Male
	W7	2	A3	KF737074	Ukraine	48.950200, 38.497600		Jul 2002	dried	Male
*Lucanus cervus turcicus*	B1	1	B1	KF737096	Bulgaria	42.162733, 27.737650		Jul 2009	ethanol	Male
	B2	1	B2	KF737098	Bulgaria	41.407800, 25.578583		Jul 2009	ethanol	Male
	B7	1	B7	KF737099	Bulgaria	42.060792, 27.977000		Jul 2009	ethanol	Male
	B9	1	B1	KF737097	Bulgaria	42.120183, 27.900405				
	F15	2	F15	KF737105	Greece	39.808333, 22.653889		Jun 2009	ethanol	Male
	F7	1	F7	KF737107	Greece	39.866667, 22.733333		Jun 2009	ethanol	
	F8	2	F7	KF737108	Greece	39.866667, 22.733333		Jun 2009	ethanol	
	F9	1	F9	KF737106	Greece	39.808333, 22.653889		Jun 2009	ethanol	Female
	F11	1	F11	KF737100	Greece	39.808333, 22.653889		Jun 2009	ethanol	Male
	F17	2	F17	KF737101	Greece	39.808333, 22.653889		Jun 2009	ethanol	
	F20	1	F20	KF737102	Greece	39.808333, 22.653889		Jun 2009	ethanol	Male
	F21	1	F21	KF737103	Greece	39.808333, 22.653889		Jun 2009	ethanol	Male
	U3	2	U3	KF737109	Turkey	41.800000, 27.950000		Jul 2009	ethanol	Male
Unknown species of *Lucanus*	H1	2	H1	KF737116	Israel	33.217100, 35.753500		Aug 2009	ethanol (after freezing)	Male
	H2	1	H2	KF737113	Israel	33.217100, 35.753500		Aug 2009	dried	Female
	H3	2	H3	KF737117	Israel	33.217100, 35.753500		Jul 2009	dried	Male
	H4	1	H4	KF737114	Israel	33.217100, 35.753500		Jul 2009	dried	Male
	H5	2	H5	KF737115	Israel	32.959600, 35.864500		1998	dried	Male
	J2†	2	J2	KF737118	Lebanon			Jul 2009	dried	Male
*Lucanus ibericus*	U6	1	U6	KF737110	Turkey	40.290300, 38.424200				
*Lucanus tetraodon provincialis*	D6	1	D6	KF737111	France	43.066700, 5.850000		Jun 2010	ethanol	Male
*Lucanus tetraodon*	X2		X2	EF487727		(Hunt et al. 2007)				
*Lucanus (Pseudolucanus) barbarossa*	SB1	1	SB1	KF737122	Spain	40.828139, -3.831811		Jul 2004	dried, later on ethanol	Male
	SB6†	2	SB6	KF737124	Spain	41.067361, -3.585322		Sep 2010	ethanol	Female
	SB7	1	SB7	KF737123	Spain	36.885000, -3.982000		May 2010	ethanol	Male
*Lucanus (Pseudolucanus) macrophyllus*	UB1†	2	UB1	KF737095	Turkey	36.501944, 33.089167		Aug 2006	dried	Male
*Dorcus parallelipipedus*		1	K1	KF737133	Montenegro					
			X3	DQ156023		(Hunt et al. 2007)				
*Lucanus formosanus*			X4	FJ606632		(Huang and Lin 2010)				
			X5	FJ606630						
			X6	FJ606628						
			X5	FJ606626						
			X5	FJ606624						
			X5	FJ606622						
			X8	FJ606583						
*Lucanus hermani*			X9	FJ606552		(Lin et al. 2011)				

**†** sequences with a maximum of seven double peaks.

### Sequencing

We first attempted to sequence the COI barcoding region with the primers developed by Folmer et al. (1994) on a subset of samples. Despite PCR optimization trials, amplification of this fragment largely failed. Instead, a 800 bp fragment of the 3’ end of the COI gene was amplified using the primer set C1-J-2183 (5’ CAACATTTATTTTGATTTTTTGG 3’) and TL2-N-3014 (5’ TCCAATGCACTAATCTGCCATATTA 3’) (Simon et al. 1994). This fragment does not overlap with the standard barcoding region. For samples O9 and V44 (Table 1) we used species-specific primers (F - 5’ GGGGCATCAGTAGACCTAGC 3’ and R – 5’ TTCAGCAGGTGGTATTAGTTGG 3’), designed from sequences on GenBank and used to PCR amplify a 1089 bp stretch of the COI gene. Reactions were performed in total volumes of 40 µl containing 5.2 µl of 10 × Taq buffer with 500 mM KCl (Fermentas, Thermo Scientific), 3.12 µl of MgCl_2_ (25 mM), 0.78 µl dNTP (10 mM), 2.08 µl of each primer (10 µM), 0.8 U Taq DNA polymerase (Fermentas, Thermo Scientific), 26.42 µl sterile distilled water. 12 µl of diluted DNA (3.5–5 ng/ µl) was added. The temperature cycle was 94 °C for 1 min, then 5 cycles of 94 °C for 1 min, 45 °C for 1 min 30 s and 72 °C for 1 min and 30 s. This was followed by 40 cycles of 94 °C for 1 min, 50 °C for 1 min 30 s and 72 °C for 1 min, and finally a single cycle at 72 °C for 5 min. PCR products were cleaned enzymatically with DNA Clean & Concentrator^TM^-5 (Zymo Research). When samples failed to amplify, mostly dried or bad quality samples, internal primers were used to allow amplification of four overlapping fragments of about 250 bp within the same 3’ end of the COI gene: LCint1 (F – 5’ CTTCGGCCACCCAGAAGT 3’ and R – 5’ TCCAGTAGGAACAGCAATRAT 3’), LCint2 (F – 5’ CGAGCCTACTTCACATCAGC 3’ and R – 5’ GCAAAAACTGCACCTATTGAAA 3’), LCint3 (F – 5’ GCTCACTTCCATTATGTACTTTCAA 3’ and R – 5’ GAGAGCCAAATGATGAAATAATGTT 3’) and LCint4 (F – 5’ CCCTGATGCCTACACCACAT 3’ and R – 5’ CCAATGCACTAATCTGCCATA 3’). PCR amplification was performed in 2.6 µl of 10x Taq buffer with 500 mM KCl, 2.08 µl of MgCl2 (25 mM), 0.39 µl dNTP (10 mM), 2.6 µl of each primer (10 µM), 0.8 U Taq DNA polymerase (Fermentas, Thermo Scientific), 9.57 µl sterile distilled water, resulting in a total volume of 20 µl to which 6 µl of diluted DNA (3.5–5 ng/ µl) was added. The PCR reaction was then conducted with the following cycle: 94 °C for 3 min, then 45 cycles of 94 °C for 45 s, 59 °C for 45 s and 72 °C for 1 min 30 s, and finally a single cycle at 72 °C for 6 min. PCR products were checked on 2% agarose horizontal gels and purified using USB® ExoSAP-IT® (Isogen Life Science). DNA sequencing was performed by a commercial company (BaseClear, Leiden, the Netherlands) or on an automatic ABI 3500 Genetic Analyzer (Applied Biosystems). Both forward and reverse primers were used except when internal primers were used for PCR, in which case sequencing was performed using the respective forward primers (except for five samples of *Lucanus (Pseudolucanus) barbarossa*, where both forward and reverse primers were used).

COI sequences available on GenBank were added. The COI sequence of *Lucanus cervus cervus* obtained by Lin et al. (2011; GenBank acc. no. FJ606555) was used as a reference for the subspecies with the highest number of specimens in this study. We selected two Asian stag beetle species, *Lucanus formosanus* Planet, 1899 and *Lucanus hermani* DeLisle, 1973, and *Dorcus parallelipipedus* (Linnaeus, 1758) (lesser stag beetle; Lucanidae) as outgroup species. Except for one available sample of the latter, the COI gene sequences of the taxa were obtained from GenBank (*Dorcus parallelipipedus*: Hunt et al. 2007; GenBank acc. no. DQ156023; *Lucanus formosanus*: Huang and Lin 2010; GenBank acc. no. FJ606632, FJ606630, FJ606628, FJ606626, FJ606624, FJ606622, FJ606583; *Lucanus hermani*: Lin et al. 2011; GenBank acc. no.: FJ606552). In the study of Hunt et al. (2007) the Dorcinae formed a sisterclade of the Lucaninae. Finally, part of the COI sequence of *Lucanus tetraodon* obtained by Hunt et al. (2007; GenBank acc. no. EF487727) was used in addition to the sequence of *Lucanus tetraodon provincialis*.

DNA sequences have been deposited in GenBank under accession numbers KF737071 to KF737133 (Table 1).

### Alignment and sequence quality control

Overall quality of the sequences was evaluated manually. Only samples with high quality chromatograms for at least 300 bp were retained for further analyses. Sequences were aligned by hand and using CLUSTALW v1.4 (Thompson et al. 1994) in BIOEDIT v7.0.0 (Hall 1999). Sequences were trimmed to 670 bases. Duplicate haplotypes were removed using DUPLICATESFINDER v1.1 (http://bioinfotutlets.blogspot.be/2009/09/duplicates-finder-java-standalone.html). We searched for potential NUMTs (nuclear mitochondrial pseudogene sequences) or heteroplasmy by manually checking for the presence of double peaks and indels, and by looking for stop codons (Song et al. 2008, Calvignac et al. 2011) using MEGA c5.01 with the implemented invertebrate mtDNA genetic code to translate the sequences (Tamura et al. 2011). We only retained sequences with a maximum of 7 polymorphic positions, which were treated as ambiguities. Finally, we constructed a Neighbour-Joining (NJ) tree with MEGA5 using 10 000 bootstraps, based on Kimura’s 2-parameter distances (K2P) (Kimura 1980). For comparison, a Bayesian inference approach (BI) was used as well. The Bayesian analysis was conducted with MRBAYES v3.1.2 (Huelsenbeck and Ronquist 2001, Ronquist and Huelsenbeck 2003) under the GTR+I+G model, simulating 4 Monte Carlo Markov Chains (MCMC) for 2 000 000 generations each. Trees were sampled every 100 generations and the first 300 000 generations were excluded as burn-in. A consensus tree was constructed with posterior probabilities. The MRBAYES analyses were carried out on the Bioportal at Oslo University (http://www.bioportal.uio.no). The GTR+I+G model used in MRBAYES is closely related to the TIM3+I+G model, which was selected by JMODELTEST v0.1.1 (Guindon and Gascuel 2003, Posada 2008) as the best-fit model under the Akaike information criterion (AIC).

### Genetic distances and nucleotide diagnostics

As K2P-distance is the most commonly used distance metric in DNA barcoding (Hebert et al. 2003), it was employed here for comparison. It allows to compare the behavior of the DNA fragment we used to the standard barcode region which is situated in the same gene. When possible, simple nucleotide diagnostics were identified for each (sub)species. If less than two simple nucleotide diagnostics were present (Sarkar et al. 2002), a compound diagnostic was detected using the algorithm of Wong et al. (2009).

## Results

### Alignment and sequence quality

Of a total of 76 samples, thirteen samples with low quality sequences were removed: five *Lucanus cervus cervus*, one *Lucanus cervus pentaphyllus*, three *Lucanus cervus turcicus* and four *Lucanus (Pseudolucanus) barbarossa*. Three sequences showed a few double peaks: one *Lucanus (Pseudolucanus) barbarossa* (SB6: 5 ambiguous sites), one *Lucanus (Pseudolucanus) macrophyllus* (UB1: 7 ambiguous sites) and one unidentified species of *Lucanus* (J2: 2 ambiguous sites) (Table 1). None exhibited indels or stop codons which are indicative of the presence of NUMTs (Buhay 2009). The remaining 63 samples and 11 sequences obtained from GenBank are listed in Table 1. The final alignment entailed 74 sequences, representing 60 haplotypes. Incomplete sequences were obtained for the following taxa: taxon H4 with 500 bp of which the reverse sequence failed and taxon J2 of which forward sequences of only the first and third smaller fragments could be produced, resulting in a total of 383 bp. Both taxa were specimens of the unidentified *Lucanus* specimens (Table 1). Likewise, the sequence of *Lucanus tetraodon* found in GenBank (named X2), was 122 bp short at the 3’ end. One other taxon, H3 (*Lucanus* sp.) missed a mere 5 bp at the 5’ end.

**Figure 1.**
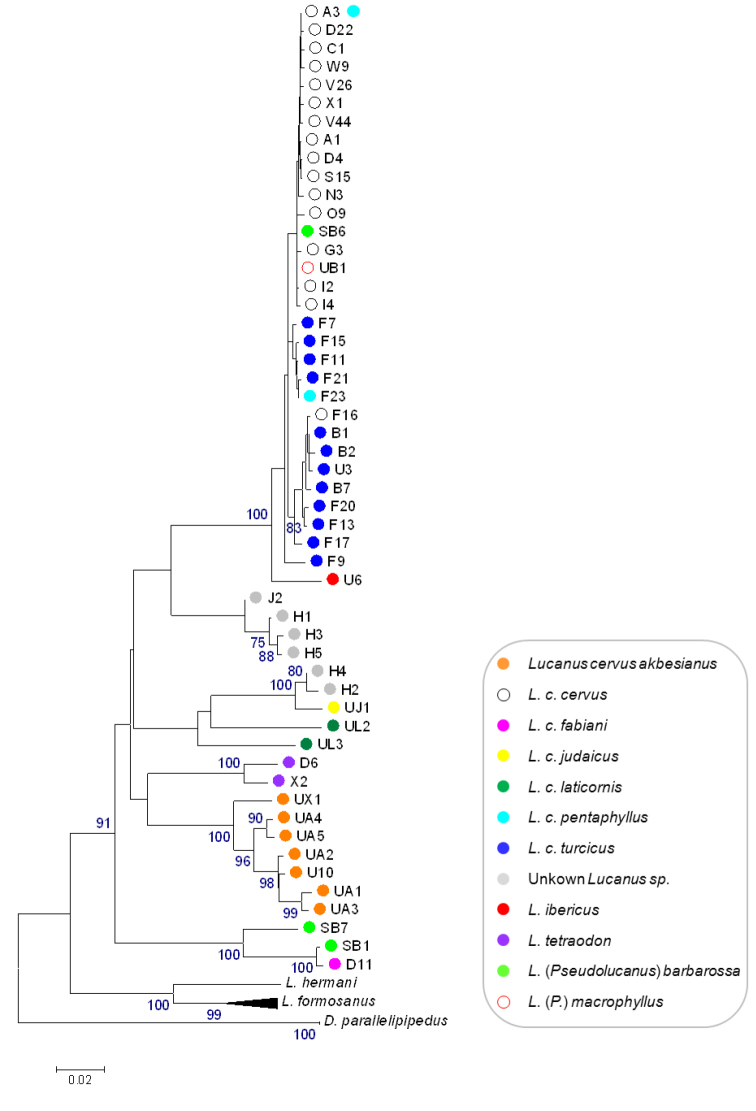
Bootstrap consensus NJ tree inferred from 10 000 replicates, with a cut off value of 70%, based on K2P-distances between 60 haplotypes of the 3’ end of the COI gene.

Both the NJ tree and the BI tree showed the same overall configuration (Figure 1 and Appendix 1, respectively) except for the position of the unidentified *Lucanus* specimens. In the NJ tree these specimens fall into two clusters with unresolved affinities (Figure 1). In the BI tree they form a single well-supported clade together with specimens identified as *Lucanus cervus judaicus* and *Lucanus cervus laticornis* (Appendix 1). The unidentified specimens fail to form a single monophyletic cluster as one subclade also includes *Lucanus cervus judaicus*. The BI tree showed *Lucanus cervus laticornis* to be monophyletic with probability 1, instead of paraphyletic as was shown in the NJ tree with bootstrap support below 70%. In both trees, several species as well as subspecies fall into distinct clades, whereas *Lucanus cervus cervus*, *Lucanus cervus turcicus*, *Lucanus cervus pentaphyllus*, *Lucanus (Pseudolucanus) macrophyllus* and *Lucanus ibericus* cluster in the same shallow clade (called the ‘*Lucanus cervus cervus* clade’ hereafter). In addition, three out of four samples of *Lucanus cervus pentaphyllus* share a haplotype with *Lucanus cervus cervus* (haplotype A3) which is the most common haplotype among *Lucanus cervus* sequences (Table 1). Within this clade *Lucanus cervus cervus*, *Lucanus cervus turcicus* and *Lucanus cervus pentaphyllus* are polyphyletic. Unexpectedly, one sample of *Lucanus (Pseudolucanus) barbarossa* and the sample of *Lucanus (Pseudolucanus) macrophyllus* are also embedded in this clade. Looking at the sequences, they only differ from haplotype A3 at their five and seven ambiguous sites, respectively. Because the two other specimens of *Lucanus (Pseudolucanus) barbarossa* form a separate clade with *Lucanus cervus fabiani*, sample SB6 is excluded from further calculations but will be discussed below.

### Genetic distances

The nucleotide composition of all the sequences was AT-rich, with 29.5% A, 35.2% T, 15.5% G and 19.7% C. There were 36.4% nucleotide sites variable and 12.1% variable amino acid sites, of which 94.3% and 77.8% were parsimony informative, respectively. When *Dorcus parallelipipedus* was excluded from the dataset, variable sites decreased to 33.3% for nucleotides and 7.2% for amino acids (94.2% and 56.2% parsimony informative, respectively). Nucleotide composition and K2P-distances calculated for each codon position are shown in Table 3.

Although specimen J2 of the unidentified specimens of *Lucanus* clustered with the other specimens of the same taxon in the NJ and BI trees, the pairwise interspecific K2P-distances with J2 differed substantially from those with H1 to H5 (comparisons with *Lucanus cervus judaicus* not included). More specifically, the minimum pairwise interspecific K2P-distance between J2 and the other unidentified taxa was 0.064 opposed to 0.087–0.095 when taking H1 to H5 into account. J2 is one of three incomplete sequences and missing information from position 179 to 399 in the sequence of J2 where several simple nucleotide diagnostics are present (Appendix 2). Therefore, this sample was removed from the dataset for subsequent analysis.

The congeneric interspecific K2P-distances between the western Palaearctic taxa and the eastern Asian species *Lucanus formosanus* and *Lucanus hermani* range from 0.156 to 0.198. Distances between taxa of *Lucanus* and *Dorcus* went from 0.211 until 0.259. K2P-distances within and between the investigated western Palaearctic taxa of *Lucanus* are shown in Table 2. As indicated by the NJ and BI trees, the taxa *Lucanus cervus cervus*, *Lucanus cervus pentaphyllus*, *Lucanus cervus turcicus* and *Lucanus (Pseudolucanus) macrophyllus* cannot be distinguished based on the COI fragment; K2P-distances range from 0 to 0.021, and all taxa are reciprocally polyphyletic. Whereas the first three subspecies of *Lucanus cervus* are distinguished solely on the basis of the number of lamellae on the antennal club, *Lucanus (Pseudolucanus) macrophyllus* is morphologically much more distinctive, resembling *Lucanus ibericus*. Although *Lucanus ibericus* is part of the *Lucanus cervus cervus* clade, it shows slightly higher K2P-distances with the other members of this clade (0.028–0.032). Note that we only had a single specimen. Moderate to relatively high within (sub)species distances were found for *Lucanus cervus laticornis* (0.085), certain specimens of the unidentified *Lucanus* sp. (max. 0.054) and *Lucanus (Pseudolucanus) barbarossa* (0.53). On the other hand, between the latter and *Lucanus cervus fabiani* a small to moderate distance exists (0.004 and 0.058). This is also the case between taxa H2 and H4 of the unknown *Lucanus* sp. and *Lucanus cervus judaicus* (K2P-distance of 0.018 and 0.016, respectively). The remaining distances between (sub)species ranged from 0.087 and 0.179.

**Table 2.** Intra- and interspecific K2P-distances for the 670 bp COI gene of western Palaearctic *Lucanus* (sub)species. NA: intraspecific K2P-distance cannot be presented because only one sample is available.

	*Lucanus cervus cervus*	*Lucanus cervus pentaphyllus*	*Lucanus cervus turcicus*	*Lucanus cervus fabiani*	*Lucanus cervus akbesianus*	*Lucanus cervus judaicus*	*Lucanus cervus laticornis*	*Lucanus ibericus*	*Lucanus tetraodon*	*Lucanus (Pseudolucanus) macrophyllus*	*Lucanus (Pseudolucanus) barbarossa*	unknown *Lucanus* sp.
***Lucanus cervus cervus***	0–0.018											
***Lucanus cervus pentaphyllus***	0–0.018	0–0.014										
***Lucanus cervus turcicus***	0.001–0.021	0.003–0.017	0–0.017									
***Lucanus cervus fabiani***	0.161–0.167	0.160–0.163	0.159–0.169	NA								
***Lucanus cervus akbesianus***	0.118–0.161	0.121–0.155	0.121–0.165	0.159–0.174	0–0.045							
***Lucanus cervus judaicus***	0.151–0.164	0.153–0.160	0.155–0.170	0.167	0.144–0.154	NA						
***Lucanus cervus laticornis***	0.134–0.160	0.134–0.155	0.132–0.164	0.162–0.165	0.135–0.150	0.089–0.094	0.085					
***Lucanus ibericus***	0.029–0.039	0.034–0.035	0.028–0.037	0.174	0.132–0.151	0.174	0.141–0.168	NA				
***Lucanus tetraodon***	0.125–0.129	0.124–0.128	0.122–0.130	0.168–0.179	0.098–0.123	0.151–0.156	0.132–0.151	0.131–0.136	0.024			
***Lucanus (Pseudolucanus) macrophyllus***	0–0.012	0–0.014	0.006–0.015	0.159	0.116–0.141	0.147	0.130–0.145	0.028	0.120–0.124	NA		
***Lucanus (Pseudolucanus) barbarossa***	0.153–0.163	0.155–0.161	0.155–0.167	0.004–0.058	0.127–0.171	0.153–0.165	0.146–0.167	0.166–0.172	0.159–0.177	0.149–0.157	0.053	
**unknown *Lucanus* sp.**	0.091–0.162	0.093–0.159	0.95–0.168	0.143–0.172	0.119–0.150	0.016–0.066	0.088–0.113	0.109–0.169	0.120–0.152	0.087–0.147	0.136–0.170	0.002–0.054

**Table 3.** Nucleotide composition and K2P-distances at each codon position of the 670 bp COI region.

	Codon position		
	1^st^	2^nd^	3^rd^
**% A**	31.4	18.9	38.2
**% T**	26.6	42.5	36.6
**% G**	25.6	16.2	4.9
**% C**	16.4	22.4	20.4
**K2P-distance**	0–0.107	0–0.032	0–0.999

These results do not show a distinct barcoding gap or other threshold to distinguish putative species, which is chiefly due to a lack of phylogenetic resolution to differentiate the said species and subspecies. If we consider the taxa of the *Lucanus cervus cervus* clade to be members of the same species, 99.4% of all intra(sub)specific comparisons showed K2P-distances below 5% and 99.8% of the pairwise inter(sub)specific distances were above 5%. Nucleotide diagnostics are listed in Appendix 2. No diagnostic combination of nucleotide positions and characters were found for the taxa of the *Lucanus cervus cervus* clade, *Lucanus ibericus* not included. As the number of species and the sample size per species are rather limited, the nucleotide diagnostics should be considered with caution.

## Discussion

The present study shows that the sequenced COI fragment could discriminate several of the investigated western Palaearctic *Lucanus* species and alleged subspecies of *Lucanus cervus*. Well differentiated species and subspecies were *Lucanus cervus akbesianus*, *Lucanus cervus laticornis* and *Lucanus tetraodon*, as well as the two eastern Asian species *Lucanus formosanus* and *Lucanus hermani*. Difficulties in molecular identification remained between *Lucanus cervus fabiani* and *Lucanus (Pseudolucanus) barbarossa*, *Lucanus cervus judaicus* and the unidentified *Lucanus* species, and between taxa of the *Lucanus cervus cervus* clade. Although thoroughly sampled within their distribution range, *Lucanus cervus cervus* and *Lucanus cervus turcicus* could not be discriminated with a barcoding approach. Likewise, three out of four samples of *Lucanus cervus pentaphyllus* possessed the most common haplotype of *Lucanus cervus cervus*. Next to introgression following recent or past hybridisation events, incomplete sorting of ancestral variation may be the reason for the polyphyletic pattern. It is not known if *Lucanus* can be infected with the endosymbiotic bacteria *Wolbachia*, which can cause mitochondrial introgression between closely related species (e.g. Whitworth et al. 2007). Nonetheless, infections with *Wolbachia* are quite common among insects, and should be taken into account (Hilgenboecker et al. 2008). However, the shift from four to five or even six lamellar segments on the antennal club is, at least in this tree of maternal inheritance, not synapomorphic among all individuals, and the number of lamellae may represent a case of parallel evolution or a phenotypically plastic trait within *Lucanus cervus*, such that *Lucanus pentaphyllus* and *Lucanus turcicus* may merely represent morphotypes of *Lucanus cervus*. This hypothesis seems less likely for *Lucanus (Pseudolucanus) macrophyllus*. Although this taxon’s haplotype only differed from the main *Lucanus cervus cervus* haplotype, A3, by its seven ambiguous sites, it has a very distinct morphology. The same can be said about *Lucanus ibericus*, which was part of the same clade, but showed higher pairwise K2P-distances (0.028–0.032) when comparing it to the other taxa of the clade. Lumping *Lucanus ibericus* and *Lucanus (Pseudolucanus) macrophyllus* together with the *Lucanus cervus* subspecies *Lucanus cervus*, *Lucanus turcicus* and *Lucanus pentaphyllus* seems therefore ill advice.

Like *Lucanus (Pseudolucanus) macrophyllus*, one sample of *Lucanus (Pseudolucanus) barbarossa*, SB6, was embedded in the *Lucanus cervus cervus* clade, opposed to the other two samples that clustered with *Lucanus cervus fabiani*. The taxa of the latter group showed K2P-distances between 0.004 and 0.058, which indicates a close relationship between *Lucanus cervus fabiani* and *Lucanus (Pseudolucanus) barbarossa*, as well as *Lucanus (Pseudolucanus) barbarossa* being very variable. High intraspecific variability could be indicative of cryptic diversity or population structure (Diptera: Meier et al. 2006; Lycaenidae: Wiemers and Fiedler 2007; Coleoptera, Nitidulidae: De Biase et al. 2012; Hemiptera, Cicadidae: Nunes et al. 2013). Despite the moderate to low genetic distance between *Lucanus (Pseudolucanus) barbarossa* and *Lucanus cervus fabiani*, these taxa are morphologically very distinct. This leaves us with either incomplete lineage sorting or introgression. Considering that both taxa have very proximate distribution ranges, introgressive hybridisation is likely. Even complete loss of the original mitochondrial genome of a species, resulting in a species with only mitochondrial genomes of the introgressed species is not unheard of (Hailer et al. 2012). Likewise, as *Lucanus cervus cervus* and *Lucanus (Pseudolucanus) barbarossa* occur sympatrically in Spain and Portugal (Méndez 2003), recent hybridisation and introgression cannot be ruled out as another or supplementary cause of the polyphyletic status of *Lucanus (Pseudolucanus) barbarossa* (Avise 2000). Because SB6 merely differed from A3 at its five ambiguous sites, it could be perceived as a shared haplotype, which would corroborate this hypothesis (e.g. Nicholls et al. 2012). *Lucanus cervus akbesianus*, *Lucanus cervus laticornis* and *Lucanus cervus judaicus* also have overlapping distributions. The former two were even sampled on the same tree in a Turkish forest (M. A. Cimaz, personal communication). In captivity, they do not seem to interbreed, which is concordant with our reporting of no shared haplotypes.

Finally, the *Lucanus* samples from Israel and Lebanon that were unidentified at the species level, seemed closely related and formed a paraphyletic clade with *Lucanus cervus judaicus*. Nevertheless, some of these samples could well be of a different species, indicated by the higher pairwise genetic distances (0.042–0.066). A detailed morphological and phylogenetic study is required here to investigate the number of species and relationship with *Lucanus cervus judaicus*.

A distinct barcoding gap was absent for several species and subspecies of *Lucanus*. This may either represent a low phylogenetic signal from the COI fragment for some relationships, a problem of basing a taxonomy on just one or a few morphological traits, or both. The use of the COI gene for barcoding purposes has had mixed results. High intraspecific variability (DeSalle et al. 2005) and closely related species (e.g. Funk and Omland 2003, Hajibabaei et al. 2006) can lead to an overlap in genetic distances, making the technique ineffective, as was shown here. In addition, NUMTs may complicate results and could cause the number of species to be overestimated (Song et al. 2008). Besides, the evolutionary history of the gene in question could be different from that of the studied species (Maddison 1997, Edwards 2009). Consequently, other or additional genes, ribosomal or nuclear, are recommended for barcoding purposes (Dupuis et al. 2012).

## Conclusions

This study revealed that while the 3’ terminus of COI contained sufficient information to resolve relationships among a number of closely related taxa, many others could not be robustly discriminated. Genotyping of additional specimens, especially of *Lucanus (Pseudolucanus) macrophyllus*, *Lucanus ibericus*, *Lucanus cervus judaicus*, *Lucanus cervus fabiani* and *Lucanus cervus laticornis*, as well as all western Palaearctic taxa is needed to fully explore COI genetic diversity and to investigate the roles of phenotypic plasticity, hybridisation and incomplete lineage sorting underlying stag beetle biodiversity and inform taxonomic investigations. We therefore see this study as a starting point for future research which should also endeavour to combine analysis of nuclear markers, such as the internal transcribed spacer (ITS) and 28S rRNA gene (e.g. Smith et al. 2007), in combination with a detailed morphological investigation, to find a useful molecular identification tool for all western Palaearctic *Lucanus* sp.

## Authors’ contributions

The work presented here was carried out in collaboration between all authors. AT, KDG, GA, PA and LB defined the subject and the design of the study. KDG designed methods and experiments in the laboratory and supervised laboratory work. KC analysed the data, interpreted results and wrote the paper. AT was responsible for collecting the samples and co-wrote the taxonomical part of the paper. JM discussed analyses. GA, ES, NMcK and PS provided five sequences and revised primarily the material and methods section and the interpretation of the results. MZ, LB and PA provided samples and co-wrote the paper, particularly the taxonomical section. DH and RM provided samples. All authors have contributed to, revised and approved the manuscript.

## Appendix 1

Consensus Bayesian tree of 60 haplotypes of the 3’ end of the COI gene. Values given by the nodes are posterior probabilities above 0.70. (doi: 10.3897/zookeys.365.5526.app1) File format: Adobe PDF file (pdf).

## Appendix 2

Nucleotide diagnostics for (sub)species or species groups according to the Neighbour-Joining and Bayesian Inference tree topology. (doi: 10.3897/zookeys.365.5526.app2) File format: Adobe PDF file (pdf).
