# Supplementary material for: Testing the performance of a fragment of the COI gene to identify western Palaearctic stag beetle species (Coleoptera, Lucanidae)
Source: Zookeys. 2013 Dec 30;(365):105–26. doi: 10.3897/zookeys.365.5526 (PMC3890674; doi:10.3897/zookeys.365.5526)

Appendix 1

Consensus Bayesian tree of 60 haplotypes of the 3' end of the COI gene. Values given by the nodes are posterior probabilities above 0.70.

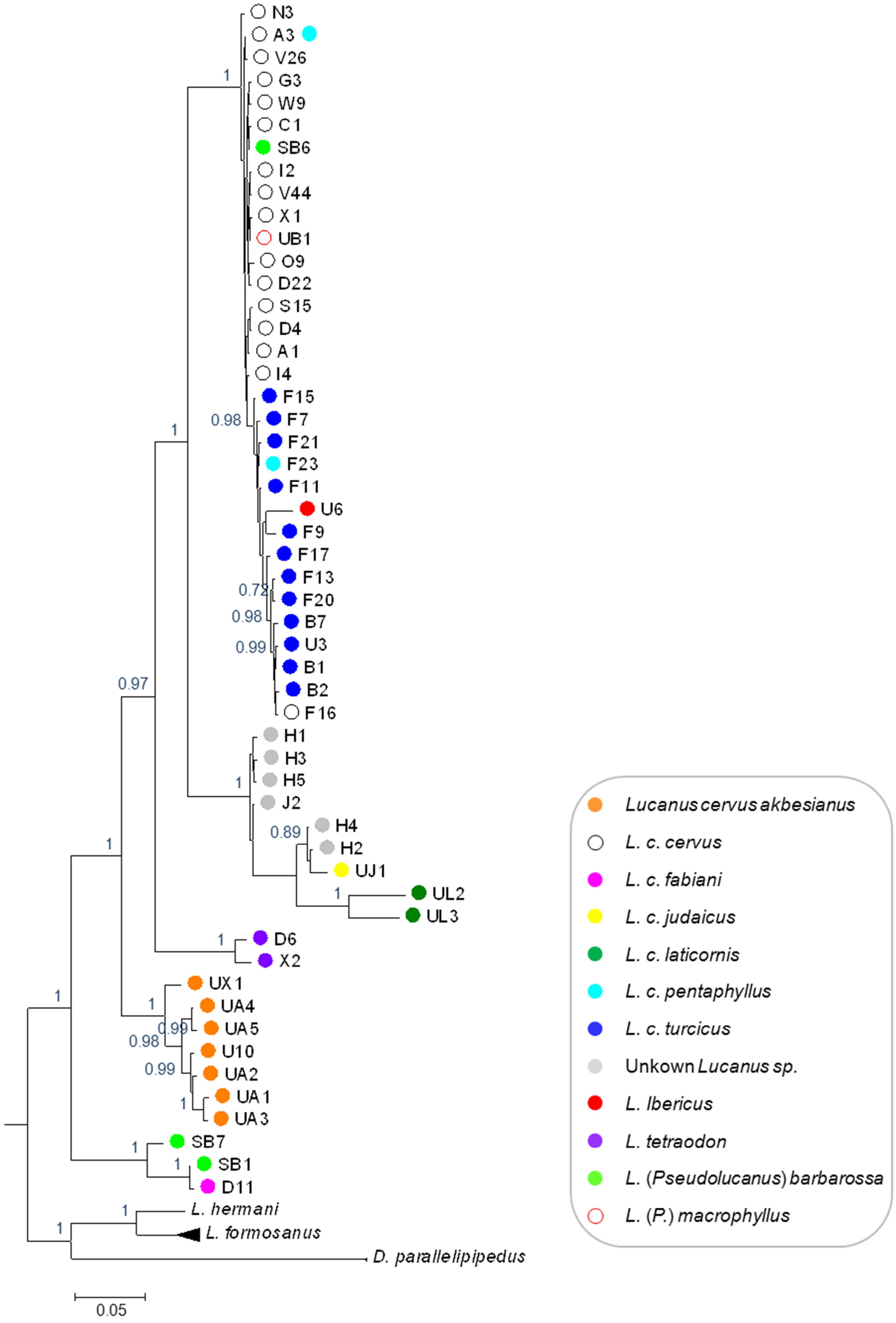

Supplement: Supplementary file 1 — Consensus Bayesian tree of 60 haplotypes of the 3’ end of the COI gene. Values given by the nodes are posterior probabilities above 0.70. (doi: 10.3897/zookeys.365.5526.app1) File format: Adobe PDF file (pdf). [file ZooKeys-365-105-s001.pdf]
