# Supplementary material for: Testing the performance of a fragment of the COI gene to identify western Palaearctic stag beetle species (Coleoptera, Lucanidae)
Source: Zookeys. 2013 Dec 30;(365):105–26. doi: 10.3897/zookeys.365.5526 (PMC3890674; doi:10.3897/zookeys.365.5526)
Supplement: Supplementary file 2 — Nucleotide diagnostics for (sub)species or species groups according to the Neighbour-Joining and Bayesian Inference tree topology. (doi: 10.3897/zookeys.365.5526.app2) File format: Adobe PDF file (pdf). [file ZooKeys-365-105-s002.pdf]

Appendix 2

Nucleotide diagnostics for (sub)species or species groups according to the Neighbour-Joining and Bayesian Inference tree topology.

**Subspecies/ species/ species group (sample size)**	**sND (cND)**	**Notes**
Lucanus cervus cervus (21) –<br>*L. c. turcicus* (13) –<br>*L. c. pentaphyllus* (4) –<br>*L.* (*P.*) *macrophyllus* (1)	(1-T + 181-C)	
L. ibericus (1)	67-C<br>(1-T + 34-G)	
Lucanus cervus cervus (21) –<br>*L. c. turcicus* (13) –<br>*L. c. pentaphyllus* (4) –<br>*L.* (*P.*) *macrophyllus* (1) –<br>*L. ibericus* (1)	199-T†<br>214-T†<br>304-C<br>319-C<br>451-G	 Ambiguous site for UB1 (*L.* (*P.*) *macrophyllus*)<br>Transversion compared to *Dorcus parallelipipedus*
L. tetraodon (2)	55-C†<br>286-C<br>355-T<br>440-C<br>523-T†	 <br>when *D. parallelipipedus* is excluded
L. c. judaicus (1)	157-G<br>328-A†<br>511-G<br>538-C†	
unknown *Lucanus* sp. (5; J2 excluded)	328-G†<br>(157-A + 253-T)	
L. c. judaicus (1) – unknown *Lucanus* sp. (5; J2 excluded)	253-T†<br>259-C<br>310-G†<br>328-A/G<br>430-C†<br>529-C†<br>574-G†	 when *D. parallelipipedus* is excluded
L. c. laticornis (2)	487-T†<br>(7-A + 571-C)	 571-C: transversion compared to *D. parallelipipedus*
L. c. akbesianus (8)	73-C†<br>130-A<br>133-T<br>250-A<br>**452-C**<br><br>523-C†<br>535-G	When *D. parallelipipedus* is excluded<br><br><br><br>sND and diagnostic amino acid (leucine) when *D. parallelipipedus* is excluded
L. c. fabiani (1)	(4-G + 331-C)	
L. (*P.*) *barbarossa* (2; SB6 excluded)	(37-T + 331-C)	
L. c. fabiani (1) – *L.* (*P.*) *barbarossa* (2; SB6 excluded)	43-G/A<br>106-C<br>313-C†<br>628-T<br>634-G†<br>661-C†	When D. parallelipipedus is excluded<br>When *D. parallelipipedus* is excluded
L. formosanus (7)	238-C/T<br>256-T†	

**Subspecies/ species/ species group (sample size)**	**sND (cND)**	**Notes**
	460-G<br>595-C<br>616-G†<br>625-T	
L. hermani (1)	4-T†<br>73-A<br>74-T<br>166-A<br>178-T<br>242-C	 <br>When *D. parallelipipedus* is excluded<br>When *D. parallelipipedus* is excluded

sND: simple nucleotide diagnostic; cND: compound nucleotide diagnostic. If less than two sNDs are present, a cND is given. NDs resulting in nonsynonymous changes were indicated in bold. NDs resulting from transversions are underlined. If both transversions and transitions could be identified at the nucleotide positions of the NDs, they are indicated with †.
